# A Multifactorial Approach for Surveillance of *Shigella* spp. and Entero-Invasive *Escherichia coli* Is Important for Detecting (Inter)national Clusters

**DOI:** 10.3389/fmicb.2020.564103

**Published:** 2020-10-19

**Authors:** Maaike J. C. van den Beld, Frans A. G. Reubsaet, Roan Pijnacker, Airien Harpal, Sjoerd Kuiling, Evy M. Heerkens, B. J. A. (Dieneke) Hoeve-Bakker, Ramón C. E. A. Noomen, Amber C. A. Hendriks, Dyogo Borst, Han van der Heide, A. M. D. (Mirjam) Kooistra-Smid, John W. A. Rossen

**Affiliations:** ^1^Infectious Disease Research, Diagnostics and Laboratory Surveillance, Centre for Infectious disease Control, National Institute for Public Health and the Environment, Bilthoven, Netherlands; ^2^Department of Medical Microbiology and Infection Prevention, University of Groningen, University Medical Center Groningen, Groningen, Netherlands; ^3^Infectious Diseases, Epidemiology and Surveillance, Centre for Infectious Disease Control, National Institute for Public Health and the Environment, Bilthoven, Netherlands; ^4^Department of Medical Microbiology, Certe, Groningen, Netherlands

**Keywords:** *Shigella*, EIEC, surveillance, genomic epidemiology, genetic characterization, phenotypic characterization, antimicrobial resistance, virulence

## Abstract

*Shigella* spp. and entero-invasive *Escherichia coli* (EIEC) can cause mild diarrhea to dysentery. In Netherlands, although shigellosis is a notifiable disease, there is no laboratory surveillance for *Shigella* spp. and EIEC in place. Consequently, the population structure for circulating *Shigella* spp. and EIEC isolates is not known. This study describes the phenotypic and serological characteristics, the phenotypic and genetic antimicrobial resistance (AMR) profiles, the virulence gene profiles, the classic multi-locus sequence types (MLST) and core genome (cg)MLST types, and the epidemiology of 414 *Shigella* spp. and EIEC isolates collected during a cross-sectional study in Netherlands in 2016 and 2017. *S. sonnei* (56%), *S. flexneri* (25%), and EIEC (15%) were detected predominantly in Netherlands, of which the EIEC isolates were most diverse according to their phenotypical profile, O-types, MLST types, and cgMLST clades. Virulence gene profiling showed that none of the isolates harbored Shiga toxin genes. Most *S. flexneri* and EIEC isolates possessed nearly all virulence genes examined, while these genes were only detected in approximately half of the *S. sonnei* isolates, probably due to loss of the large invasion plasmid upon subculturing. Phenotypical resistance correlated well with the resistant genotype, except for the genes involved in resistance to aminoglycosides. A substantial part of the characterized isolates was resistant to antimicrobials advised for treatment, i.e., 73% was phenotypically resistant to co-trimoxazole and 19% to ciprofloxacin. AMR was particularly observed in isolates from male patients who had sex with men (MSM) or from patients that had traveled to Asia. Furthermore, isolates related to international clusters were also circulating in Netherlands. Travel-related isolates formed clusters with isolates from patients without travel history, indicating their emergence into the Dutch population. In conclusion, laboratory surveillance using whole genome sequencing as high-resolution typing technique and for genetic characterization of isolates complements the current epidemiological surveillance, as the latter is not sufficient to detect all (inter)national clusters, emphasizing the importance of multifactorial public health approaches.

## Introduction

Shigellosis is an enteric disease, caused by the species *Shigella dysenteriae, Shigella flexneri, Shigella boydii*, and *Shigella sonnei.* Entero-invasive *Escherichia coli* (EIEC) is a pathotype of *E. coli* with similar pathogenicity as *Shigella* spp., and they are genetically similar (Kaper et al., 2004; Pettengill et al., 2015). They can only be distinguished by combining a large amount of classical phenotypic tests with classical O-serotyping or *in silico* analyses of O-antigen genes. However, none of those methods can distinguish all isolates accurately (Chattaway et al., 2017; van den Beld et al., 2018).

In Netherlands, major risk factors for contracting an infection with *Shigella* spp. or EIEC are traveling and, for men, sexual contacts with other men (Pijnacker et al., 2017; van den Beld et al., 2019b). Other countries reported that shigellosis amongst men who have sex with other men (MSM) is often associated with high-risk sexual behavior and co-infection with human immunodeficiency virus (HIV; Hoffmann et al., 2013; Mohan et al., 2018; Wu et al., 2019). Genomic epidemiology studies based on whole genome sequencing (WGS) demonstrate that MSM-associated clusters of *S. sonnei* and *S. flexneri* often coincide with multi-resistance against antimicrobials (Hoffmann et al., 2013; Baker et al., 2015, 2018b; Bowen et al., 2016; Mook et al., 2016; Ingle et al., 2019). Antimicrobial resistance (AMR) of *Shigella* spp. is encoded on multiple mobile genetic elements (MGE) that can be horizontally transferred, including plasmids such as spA or pCERC1, and chromosomal integrons such as the SRL-MDRE island and ln2 and the transposon tn7 (Holt et al., 2012; Baker et al., 2015, 2018a). In the United Kingdom (UK) and France, it was demonstrated that MSM lineages of *S. sonnei* and *S. flexneri* are associated with the presence of the pKSR100 plasmid that contains genes involved in beta-lactam and azithromycin resistance (Baker et al., 2018b). Next to these horizontally transmitted AMR transferred by MGE, vertically transferred chromosomal point mutations mainly conferring resistance to quinolones can be present (Chung The et al., 2016; Ingle et al., 2019).

All species of *Shigella* and EIEC display a virulent phenotype by which human epithelial cells are invaded and disrupted (Kaper et al., 2004; Mattock and Blocker, 2017). Virulence genes are encoded on chromosomal pathogenicity islands, SHI-1, SHI-2, and SHI-3, the latter specifically for *S. boydii* (Mattock and Blocker, 2017). Additionally, *Shigella* spp. and EIEC possess a large invasion plasmid (pINV) that encodes virulence genes, including the Type III secretion system (T3SS) that is important for invasion, and the T3SS effectors that are secreted into host cells to induce a regulated inflammation in the human host, beneficiary for the bacteria (Lima et al., 2015; Mattock and Blocker, 2017). Different species of *Shigella* are known to produce Shiga-toxin, present in phage P27-, or POC-J13-related prophage sequences on the chromosome (Gray et al., 2015; Mattock and Blocker, 2017). One study was performed in which the presence of virulence genes was linked to certain phylogenetic clades of EIEC (Hazen et al., 2016). Many studies into virulence genes of *Shigella* spp. were performed, however, they were never associated with certain phylogenetic lineages to our knowledge.

Since 2012, the global population structure based on WGS was unraveled for *S. dysenteriae* (Njamkepo et al., 2016), *S. flexneri* (Connor et al., 2015), and *S. sonnei* (Holt et al., 2012) identifying global lineages. Later, the presence or absence of specific *S. flexneri* and *S. sonnei* global lineages in the United States of America (Abelman et al., 2019), Latin America (Baker et al., 2017), Australia (Ingle et al., 2019), United Kingdom, and France (Baker et al., 2018a,b) were confirmed.

In Netherlands, as in many other countries, infections with *Shigella* spp. are notifiable by law, while infections with EIEC are not. Epidemiological surveillance of individual shigellosis patients is in place as regulation for the control of shigellosis, and contact tracing is performed in all cases. However, no active laboratory surveillance is employed; consequently, the population structure for *Shigella* spp. and EIEC isolates circulating in Netherlands is not known.

During 2016 and 2017, a cross-sectional study was conducted, and throughout this study 15 participating Dutch medical microbiological laboratories (MMLs) sent all their *Shigella* spp. and EIEC isolates to the study group. All isolates were thoroughly characterized, both phenotypically and genotypically, in conjunction with epidemiological data of the patients that were infected. This is the first study that assessed the genomic epidemiology of *S. flexneri, S. sonnei*, and EIEC isolates in Netherlands within the perspective of the global populations. Furthermore, it is the first study that performed virulence gene profiling in the context of phylogenetic clustering of isolates.

## Materials and Methods

### Isolates, Phenotypic Characterization, Antimicrobial Resistance, and Epidemiological Data Collection

A total of 414 EIEC and *Shigella* spp. isolates were collected by 15 MMLs in Netherlands that were participating in the cross-sectional Invasive Bacteria *E. coli-Shigella* study (IBESS) performed in 2016–2017 (van den Beld et al., 2019b). All isolates were thoroughly characterized, both phenotypically, and genotypically. Identification and *Shigella* and *E. coli* O-serotyping of isolates was performed as described before (van den Beld et al., 2018). In short, it was based on an identification as either *E. coli* or *Shigella* using matrix-assisted laser desorption/ionization- time of flight (MALDI-TOF) mass spectrometry, and a positive PCR targeting the *ipaH* gene, followed by profiling of established phenotypical and serological features. Isolates were called provisional *Shigella* if the species and serotype could not be determined due to auto-agglutination or inconclusive combinations of antisera. Furthermore, isolates were called provisional *Shigella* if a serotype could be assigned, but the results of the phenotypical tests deviated from those of the serotype-specific tests. Overall, phenotypic properties of *S. flexneri, S. sonnei*, and EIEC were compared. To gather the epidemiological data linked to the isolates, patients were contacted by infectious disease nurses from the public health services Groningen and Amsterdam to collect information on demographics, travel history, sexual behavior, and indicators for high-risk sexual behavior such as HIV status, presence of other sexually transmitted infections (STI), and the use of pre-exposure prophylaxis (PrEP) using a standardized survey by telephone (van den Beld et al., 2019b).

### Ethics Approval and Consent to Participate

The IBESS-study was registered as an observational study under number 23481 in the Dutch Trial Register. Patients were informed about the study and subjected to a single survey after their consent, to collect additional clinical and epidemiological data. In case of minors, one of the parents or caretakers was asked to participate in the survey. The medical ethics review board (METC) in Utrecht, Netherlands, stated that this study was not subject to “medical research with human subjects” laws (protocol number 15-414/C). Data handling complied with the Dutch Personal Data Protection Act and with the EU General Data Protection Regulation.

### Sequencing and Data Preparation

Based on the species designations and availability of patient data, 348 of 414 isolates (Table 1) were selected for WGS using Illumina^®^ technology as described previously (van den Beld et al., 2018). Resulting raw reads were processed with an in-house assembly pipeline,^1^ consisting of quality assessment using FastQC v. 0.11.8 (Ewels et al., 2016), and MultiQC v. 1.7 (Brown et al., 2017), read trimming using ERNE v. 2.1.1 (Del Fabbro et al., 2013), contamination filtering using CLARK v. 1.2.5.1 (Ounit and Lonardi, 2016), assembly using SPAdes v. 3.10.0 (Bankevich et al., 2012), and assembly quality assessment using QUASTv. 4.4 (Gurevich et al., 2013). Completeness and contamination of assemblies were checked using CheckM v. 1.0.11 (Parks et al., 2015; taxonomy_wf: genus “*Shigella”*), draft genomes with good quality, and completeness higher than 99% and contamination lower than 2% were used in further analysis. All sequences were submitted to the Sequence Read Archive (SRA) under study number PRJEB32617.

**TABLE 1:** Reference sequences used for detection of *Shigella* virulence operons/genes.

**Gene/operon**	**Origin**	**Accession number**
**SHI-1 PAI**		
*sigA*	*S. flexneri* 2a str. 301	NC_004337.2
*pic*	*S. flexneri* 2a str. 301	NC_004337.2
*set*	*S. flexneri* 2a str. 301	NC_004337.2
**SHI-2 PAI**		
*iucA*	*S. flexneri* 2a str. 301	NC_004337.2
*iucB*	*S. flexneri* 2a str. 301	NC_004337.2
*iucC*	*S. flexneri* 2a str. 301	NC_004337.2
*iucD*	*S. flexneri* 2a str. 301	NC_004337.2
*iutA*	*S. flexneri* 2a str. 301	NC_004337.2
*shiA*	*S. flexneri* 2a str. 301	NC_004337.2
*shiB*	*S. flexneri* 2a str. 301	NC_004337.2
*shiD*	*S. flexneri* 2457T	AE014073.1
*shiE*	*S. flexneri* 5 str. 8401	CP000266.1
**T3SS machinery (pINV)**		
*mxi-spa* operon *(mxiG-spa)*	*S. flexneri* 2a str. 301, virulence plasmid pCP301	AF386526.1
**T3SS effectors (pINV)**		
*ipa-ipg* operon	*S. flexneri* 2a str. 301, virulence plasmid pCP301	AF386526.1
*virA*	*S. flexneri* 2a str. 301, virulence plasmid pCP301	AF386526.1
*ospB*	*S. flexneri* 2a str. 301, virulence plasmid pCP301	AF386526.1
*ospC1*	*S. flexneri* 2a str. 301, virulence plasmid pCP301	AF386526.1
*ospC3*	*S. flexneri* 2a str. 301, virulence plasmid pCP301	AF386526.1
*ospD3 (sen)*	*S. flexneri* 2a str. 301, virulence plasmid pCP301	AF386526.1
*ospE1*	*S. flexneri* 2a str. 301, virulence plasmid pCP301	AF386526.1
*ospE2*	*S. flexneri* 2a str. 301, virulence plasmid pCP301	AF386526.1
*ospF*	*S. flexneri* 2a str. 301, virulence plasmid pCP301	AF386526.1
*ospG*	*S. flexneri* 2a str. 301, virulence plasmid pCP301	AF386526.1

### Antimicrobial Resistance

Phenotypic AMR profiling was performed by participating MMLs of the IBESS study using their own, undisclosed routine diagnostic protocols. *In silico* resistance profiling was performed to assess the presence of antimicrobial resistance genes (ARGs) and chromosomal point mutations. For this purpose, the ResFinder and PointFinder databases and scripts were obtained from the Center for Genomic Epidemiology (CGE) repositories at Bitbucket.^2^ These scripts were integrated into a local pipeline script for batch execution and were executed using the default analysis settings and the applicable databases. Logistic regression models were used to associate the presence of ARGs with phenotypic resistance. Intermediate phenotypes were not considered. Associations were expressed as odds ratios (OR) with corresponding 95% confidence intervals (CI).

### MLST and cgMLST Analysis

Classical MLST and a Core genome multi-locus sequence typing (cgMLST) analyses were performed with Ridom SeqSphere^+^, version 3.5.1 (Ridom^©^ GmbH, Münster, Germany). The *E. coli* Warwick MLST scheme, curated by MLST databases of the University of Warwick (Wirth et al., 2006) and the *E. coli* cgMLST genotyping scheme based on the EnteroBase *Escherichia/Shigella* cgMLST v1 scheme were used. For global context, isolates representing *S. sonnei* lineages I, II, III, IV, V, and the subclades of lineage III; IIIa, global III, orthodox Jewish communities associated (OJCA) III, Central Asia associated III, and MSM clades 1 to 4 were added to the cgMLST (Holt et al., 2012; Chung The et al., 2016; Baker et al., 2017, 2018b). For *S. flexneri*, isolates were included that represent phylogenetic groups PG1 to PG7, including the PG3 major and minor MSM subclade (Baker et al., 2018b) and *S. flexneri* 3a MSM sublineages A, B, C, and Asia and Africa associated sublineages (Baker et al., 2015). For EIEC, no global population studies were performed, but isolates representing 3 different STs and 9 serotypes encountered in England during 2005–2016 were included (Cowley et al., 2018). Details about used reference genomes were summarized in Supplementary Table 1. Trees were inferred based on cgMLST in Ridom SeqSphere^+^, and visualized using iTOL v4.3.2 (Letunic and Bork, 2019).

### Virulence Profiling

For assessment of virulence genes, the VirulenceFinder database for *E. coli* virulence genes was used from the CGE (Joensen et al., 2014). For *Shigella* virulence, genes present in the SHI-1, SHI-2 pathogenicity islands as well as the genes responsible for the T3SS machinery and effectors were used as reference (Table 1). Reference genes were indexed based on gene name and accession code obtained from the National Center for Biotechnology Information (NCBI), to make a nucleotide comparison in a local alignment. Both indexing of the reference genes and alignment with the isolates were facilitated by the command line BLAST application, used with default settings and identity cut-offs of 70% (Camacho et al., 2009).

## Results

### Phenotypic Characterization

414 isolates were collected during 2 years from 411 patients. Three of these patients suffered from an infection with two species. From those 414 isolates, 204 were isolated in 2016 and 210 were isolated in 2017. Both years displayed a comparable species distribution (χ2, *p* = 0.69). In total, 232 isolates were *S. sonnei*, 104 *S. flexneri*, 64 EIEC, 10 provisional *Shigella*, 3 *S. boydii*, and one isolate was either EIEC or *S. flexneri*, the distinction could not be made (Table 2). No *S. dysenteriae* was identified.

**TABLE 2:** Isolates and their identification, sequence status, and patient data availability.

**Species**	***n* total**	***n* (%) sequenced^a^**	***n* (%) patient data available**	***n* (%) sequenced and data**
*S. dysenteriae*	0	0	0	0
*S. flexneri*	104	87 (84)	79 (76)	79 (76)
*S. boydii*	3	2 (67)	2 (67)	2 (67)
*S. sonnei*	232	190 (82)	168 (72)	168 (72)
Provisional *Shigella*	10	6 (60)	8 (80)	5 (50)
EIEC	64	62 (97)	33 (52)	32 (50)
EIEC/*S. flexneri*	1	1 (100)	1 (100)	1 (100)
Total	414	348 (84)	291 (70)	287 (69)

*^*a*^All EIEC isolates were sequenced, except for two that were not available anymore. Other selection for sequencing was based on definitive species identification and data availability.*

For *S. flexneri*, serotype 2a was mostly identified (51%), followed by serotype 6 (12%), 1c (7%), 3a (7%), 1b (5%), 4av (3%), Xv (3%), Y (3%), 3b (2%), Yv (2%), and 1a (1%). For 6% of *S. flexneri* isolates, the serotype could not be determined due to undescribed combinations of reactions with antisera.

Of the 64 EIEC isolates, 24 (38%) were negative for *E. coli* O1 – O188 antisera. The other 40 isolates were distributed over 16 different O-types, of which 32 (50%) EIEC isolates had O-types that were described as EIEC-associated before (O42, O96, O121, O124, O135, O136, O143, O159, and O164). Additionally, 8 (13%) of EIEC isolates had O-types that were not described as EIEC-associated before (O8, O10, O17, O48, O73, O109, and O141). Results from phenotypic tests for *S. flexneri, S. sonnei*, and EIEC are summarized in Table 3.

**TABLE 3:** Phenotypic traits of *S. sonnei*, *S. flexneri* and EIEC, in percentage of positives.

**Phenotypic trait**	***S. sonnei* (*n* = 232)**	***S. flexneri* (*n* = 104)**	**EIEC (*n* = 64)**
Motility^a^	0	0	30
LDC^a^	0	0	45
ODC	98	0	41
ADH	2	5	6
Esculin^a^	0	0	8
Indole	0	16	77
Gas from D-glucose	0	0	72
Indole + gas from D-glucose^a^	0	0	59
ONPG	90	1	89
**Fermentation of:**			
D-glucose	99	99	100
Lactose	2	1	69
D-sucrose	2	0	44
D-xylose	39	8	84
D-mannitol	81	96	97
Dulcitol	0.4	0	34
D-sorbitol	0.4	5	88
Salicin^a^	0	0	5
D-trehalose	100	82	97
D-raffinose	0.4	8	45
Glycerol	9	3	50

*^*a*^Tests used for distinction of *Shigella spp.* from *E. coli* that are by definition negative for *Shigella spp.**

### Antimicrobial Resistance

A total of 180 out of 248 *Shigella* spp. and EIEC isolates (73%) were phenotypical resistant to co-trimoxazole, 49 out of 264 (19%) were resistant to ciprofloxacin, and 34 (14%) were resistant to both. *In silico* determination of azithromycin resistance genes *erm(B)* and *mphA* was performed, in 30 (9%) out of all 348 genomes *erm(B)* was detected, in 37 (11%) *mphA*, and in 29 (8%) both genes were detected. The detected ARGs and their association with phenotypic resistance are shown in Table 4. Presence of blaTEM-1b, as well as the presence ≥1 *bla* genes were significantly associated with phenotypic resistance against ampicillin. Furthermore, blaTEM-1b, blaOXA-1, and the presence of ≥1 *bla* genes were significantly associated with phenotypic resistance against amoxicillin/clavulanic acid (Table 4). Only one of the isolates phenotypically tested resistant to piperacillin/tazobactam, but no *bla* genes were detected in this isolate. Of the isolates that were phenotypically resistant to 3rd generation cephalosporins, cefotaxime, and ceftazidime, respectively, 100% and 86% contained one of the *bla-CTX-M* genes or the *blaDHA-1* gene (Table 4). Phenotypical resistance to aminoglycosides gentamicin and tobramycin was not associated with the presence of *aac(3)-IId* or *aph(3)-Ia* genes. Other ARGs that confer resistance to gentamicin or tobramycin were not detected. Phenotypical resistance to ciprofloxacin was significantly associated with three chromosomal point mutations that are known to confer resistance in the *gyrA* and *par* genes (Chung The et al., 2016; Sadouki et al., 2017). All isolates that displayed resistance to ciprofloxacin, except one *S. sonnei* isolate, possessed two or more chromosomal point mutations, while the presence of plasmid-mediated *qnr* genes or the presence of one chromosomal point mutation was not associated with the resistant phenotype. Phenotypic resistance to trimethoprim perfectly correlated with the presence of one or more *dfrA* genes (Table 4). All isolates that were phenotypically resistant to co-trimoxazole, except one EIEC isolate, had one or more *dfrA* genes, and the presence of one or more *dfrA* genes combined with one or more *sul* genes was also significantly associated with co-trimoxazole resistance (Table 4). None of the ARGs were exclusively found in restricted periods.

**TABLE 4:** Phenotypic resistance of isolates, and the presence of associated antimicrobial resistance genes.

	**Resistant**		**Sensitive**		**OR (95% CI)^a^**		**Resistant**		**Sensitive**		**OR (95% CI)^a^**
	**phenotype**		**phenotype**				**phenotype**		**phenotype**		
	***n***	**%**	***n***	**%**			***n***	**%**	***n***	**%**	
Ampicillin (*n* = 241)	109	45	132	55		Gentamicin (*n* = 243)	17	7.0	226	93.0	
*blaTEM-1b*	46	42.2	2	1.5	47.5 (11.2–201-8)	*aac(3)-IId*	1	5.9	0	0	
*blaTEM-1c*	2	1.8	0	0		*aph(3)-Ia*	1	5.9	0	0	
*blaTEM-30*	1	0.9	0	0		≥*1 of aac or aph gene*	2	11.8	0	0	
*blaDHA-1*	1	0.9	0	0		Tobramycin (*n* = 238)	15	6.3	223	93.7	
*blaOXA-1*	55	50.5	0	0		*aac(3)-IId*	1	6.7	0	0	
*blaCTX-M-15*	10	9.2	0	0		*aph(3)-Ia*	1	6.7	0	0	
*blaCTX-M-32*	1	0.9	0	0		≥*1 of aac or aph gene*	2	13.3	0	0	
*blaCTX-M-55*	2	1.8	0	0		Ciprofloxacin (*n* = 264)	49	18.6	215	81.4	
≥*1 of bla genes*	106	97.2	2	1.5	2296.7 (376.8–13998.4)	*qnrB19*	0	0	12	5.6	
Amoxicillin/clavulanic acid (*n* = 227)	57	25	170	75		*qnrB4*	0	0	1	0.5	
*blaTEM-1b*	19	33.3	23	13.5	3.2 (1.6–6.5)	*qnrS1*	4	8.2	12	5.6	
*blaTEM-1c*	0	0	2	1.2		*gyrA S83A^b^*	0	0	1	0.5	
*blaTEM-30*	1	1.8	0	0		*gyrA S83L^b^*	48	98.0	36	16.7	238.7 (31.9–1785.5)
*blaDHA-1*	0	0	1	0.6		*gyrA D87G^b^*	28	57.1	2	0.9	142.0 (31.6–638.3)
*blaOXA-1*	39	68.4	14	8.2	24.1 (11.0–52.8)	*gyrA D87Y^b^*	2	4.1	3	1.4	
*blaCTX-M-15*	2	3.5	2	1.2		*gyrA D87N^b^*	18	36.7	0	0	
*blaCTX-M-32*	1	1.8	0	0		*parC S80I^b^*	48	98.0	1	0.5	10272.0 (631.3–167142.7)
*blaCTX-M-55*	1	1.88	0	0		*parE S458A^b^*	12	24.5	0	0	
≥*1 of bla genes*	56	98.2	39	22.9	188.1 (25.2–1403.1)	≥*1 of qnr genes*	4	8.2	25	11.6	
Piperacillin/Tazobactam (*n* = 227)	1	0.4	226	99.6		*1 point mutation*	1	2.0	40	18.6	
*blaTEM-1b*	0	0	43	19.0		≥*2 of point mutations*	48	98.0	1	0.5	10272.0 (631.3–167142.7)
*blaTEM-1c*	0	0	2	0.9		≥*1 of genes/mutations*	49	100	64	29.8	
*blaTEM-30*	0	0	1	0.4		Trimethoprim (*n* = 181)	157	86.7	24	13.3	
*blaOXA-1*	0	0	49	21.7		*dfrA1*	131	83.4	2	8.3	55.4 (12.3–250.2)
*blaCTX-M-15*	0	0	9	4.0		*dfrA14*	21	13.4	1	4.2	
*blaCTX-M-32*	0	0	1	0.4		*dfrA17*	13	8.3	0	0	
*blaCTX-M-55*	0	0	1	0.4		*dfrA7*	3	1.9	0	0	
≥*1 of bla genes*	0	0	97	42.9		*dfrA8*	1	0.6	0	0	
Cefotaxime (*n* = 241)	13	5.4	228	94.6		≥*1 of dfrA genes*	157	100	3	12.5	
*blaCTX-M-15*	10	76.9	0	0		Trimethoprim/sulfonamide (cotrimoxazole; *n* = 248)	180	72.6	68	27.4	
*blaCTX-M-32*	1	7.7	0	0		*Sul1*	24	13.3	1	1.5	10.3 (1.4–77.8)
*blaCTX-M-55*	2	15.4	0	0		*Sul2*	166	92.2	8	11.8	88.9 (35.5–222.6)
≥*1 of blaCTX-M genes*	13	100	0	0	n.c.^c^	*Sul3*	1	0.6	0	0	
Ceftazidime (*n* = 242)	7	2.9	235	97.1		*dfrA1*	143	79.4	36	52.9	3.4 (1.9–6.2)
*blaDHA-1*	1	14.3	0	0		*dfrA14*	30	16.7	0	0	
*blaCTX-M-15*	3	42.9	4	1.7	43.3 (7.2–260.4)	*dfrA17*	18	10.0	1	1.5	
*blaCTX-M-32*	1	14.3	0	0		*dfrA5*	1	0.6	0	0	
*blaCTX-M-55*	1	14.3	0	0		*dfrA7*	5	2.8	0	0	
≥*1 of blaDHA/CTX-M*	6	85.7	4	1.7	346.5 (33.5–3584.1)	*dfrA8*	1	0.6	0	0	
						≥*1 of sul genes*	172	95.6	9	13.2	140.9 (52.0–382.1)
						≥*1 of dfrA genes*	179	99.4	37	54.4	150.0 (19.8–1133.4)
						≥*1 of dfrA and* ≥ *1 sul genes*	172	95.6	3	4.4	465.8 (119.9–1810.0)

*^*a*^If significant, odds ratio (OR) with 95% confidence interval (CI) are displayed. ^*b*^Chromosomal point mutation at position n. ^*c*^Non-calculable because it perfectly predicts phenotypic resistence.*

### MLST and cgMLST Analysis

With classical MLST typing, most *S. sonnei* isolates (96%) were ST152, most *S. flexneri* serotype 1 to 5 isolates (91%) were ST245, and all *S. flexneri* serotype 6 isolates were ST145. In contrast, STs of EIEC isolates were diverse and distributed over 18 known STs, and 5 unknown STs, the latter all consisting of different allele combinations. Of the 18 known STs, 12 were assigned to single EIEC isolates, while ST6 comprises 13 EIEC isolates (21%), ST99 9 isolates (15%), ST4267 8 EIEC isolates (13%), ST245 and ST270 6 (10%) EIEC isolates each, and ST311 3 isolates (5%).

**FIGURE 1:**
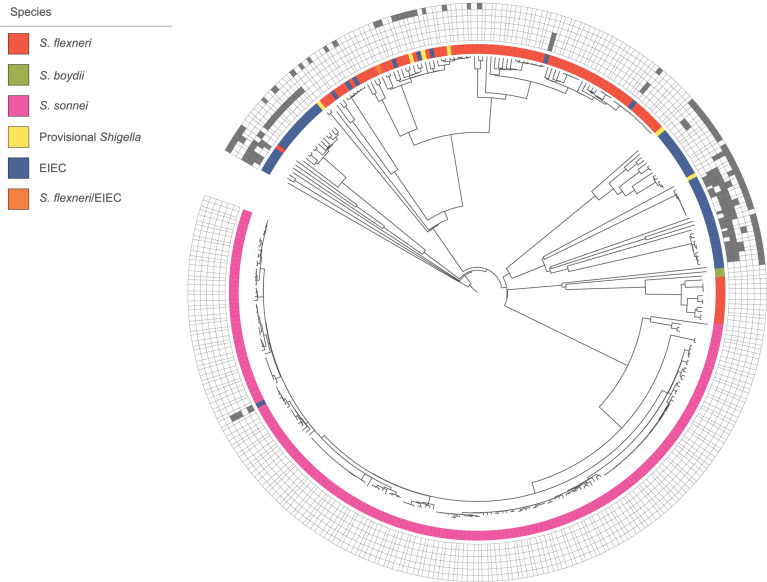
Core genome MLST tree of all isolates with species designations. 348 isolates, distance based on comparing 2315 alleles using the Enterobase *Escherichia/Shigella* cgMLST v1 scheme. Missing values are an own category. Gray squares = results of decisive phenotypic tests or serology, box with border only = negative, and filled square = positive. Phenotypic/serologic tests from inner to outer ring: motility, lysine decarboxylase, combination of gas and indole, esculin, salicin fermentation, and inconclusive S*higella* serology.

**FIGURE 2:**
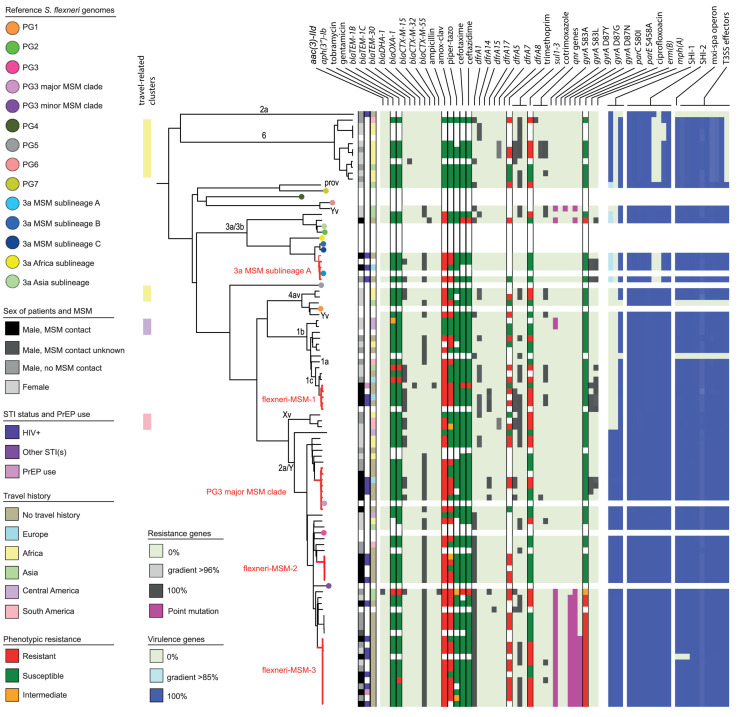
Core genome MLST tree of *S. flexneri*, including context isolates. 101 isolates, distance based on comparing 2315 alleles using the Enterobase *Escherichia/Shigella* cgMLST v1 scheme. Missing values are an own category. Red text = MSM-associated clusters. Black text = serotype; prov = provisional *Shigella*. *Qnr* genes left to right = *qnrB19*, *qnrB4*, *qnrS1*; SHI-1 left to right = *sigA, pic, set*; SHI-2 left to right = *iucA, iucB, iucC, iucD, iutA, shiA, shiB, shiD, shiE*; and T3SS effectors left to right = *ipa-ipg* operon, *virA, ospB, ospC1, ospC3, ospD3, ospE1, ospE2, ospF, ospG.* Further features are explained in the legend within the figure.

**FIGURE 3:**
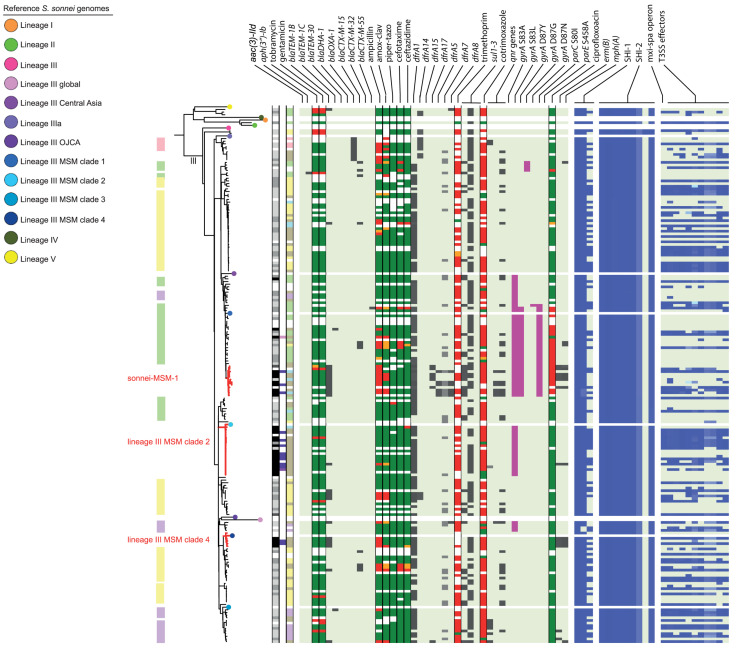
Core genome MLST tree of *S. sonnei*, including context isolates. 203 isolates, distance based on comparing 2315 alleles using the Enterobase *Escherichia/Shigella* cgMLST v1 scheme. Missing values are an own category. Red text = MSM-associated clusters. Black text = serotype; prov = provisional *Shigella*. *Qnr* genes left to right = *qnrB19*, *qnrB4*, *qnrS1*; SHI-1 left to right = *sigA, pic, set*; SHI-2 left to right = *iucA, iucB, iucC, iucD, iutA, shiA, shiB, shiD, shiE;* and T3SS effectors left to right = *ipa-ipg* operon, *virA, ospB, ospC1, ospC3, ospD3, ospE1, ospE2, ospF, ospG.* Further features are explained in the legend within Figure 2.

**FIGURE 4:**
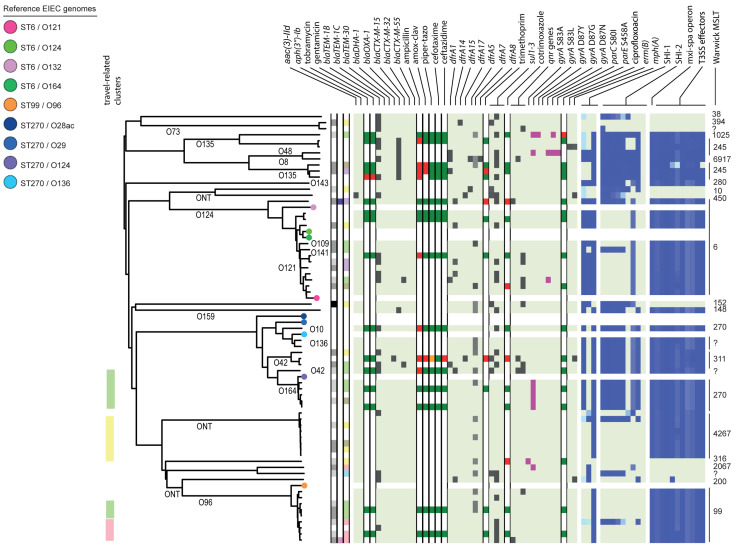
Core genome MLST tree of EIEC, including context isolates. 71 isolates, distance based on comparing 2315 alleles using the Enterobase *Escherichia/Shigella* cgMLST v1 scheme. Missing values are an own category. Red text = MSM-associated clusters. Black text = serotype; prov = provisional *Shigella*. *Qnr* genes left to right = *qnrB19*, *qnrB4*, *qnrS1*; SHI-1 left to right = *sigA, pic, set*; SHI-2 left to right = *iucA, iucB, iucC, iucD, iutA, shiA, shiB, shiD, shiE;* and T3SS effectors left to right = *ipa-ipg* operon, *virA, ospB, ospC1, ospC3, ospD3, ospE1, ospE2, ospF, ospG.* Further features are explained in the legend within Figure 2.

In the cgMLST tree including all isolates, most of the genomes clustered according to their species, although also clusters with mixed species were formed (Figure 1). Three separate cgMLST trees were created for *S. flexneri, S. sonnei* and EIEC including context isolates. From 291 of the 348 (84%) sequenced genomes, data about patient demographics, travel history, sexual behavior, and indicators for high-risk sexual behavior as HIV status, presence of other STIs and the use of PrEP was collected and depicted in the cgMLST trees (Figures 2–4).

Based on cgMLST, *S. flexneri* and EIEC isolates clustered predominantly according to their serotype or O-types, respectively, (Figures 2, 4). Although for EIEC isolates, two clusters had O135 interspersed by EIEC with O-types O8 and O48 (Figure 4). Additionally, isolates with ST270 and O-type O164 clustered with reference EIEC ST270/O124.

### MSM Associated Clusters

Although for EIEC MSM associated clusters were not identified, five *S. flexneri* clusters and three *S. sonnei* clusters were associated with MSM (Figures 2–4). Four of these MSM clusters were described in previous publications, i.e., *S. flexneri* 3a MSM sublineage A, *S. flexneri* PG3 major MSM subclade (Baker et al., 2015, 2018a; Figure 2), *S. sonnei* lineage III MSM clade 2 and *S. sonnei* lineage III MSM clade 4 (Baker et al., 2018b; Figure 3). The additional four clusters were labeled flexneri-MSM-1, flexneri-MSM-2, flexneri-MSM-3, and sonnei-MSM-1. Four out of the five *S. flexneri* MSM clusters consisted of *S. flexneri* serotypes 2a/Y and 3a that were earlier described within MSM lineages, while flexneri-MSM-1 contained only *S. flexneri* serotype 1c. The clusters flexneri-MSM- 1, flexneri-MSM-2, and *S. sonnei* lineage III MSM clade 4 consisted of only MSM, while percentages of reported MSM in cluster *S. flexneri* 3a MSM sublineage A (67%), *S. flexneri* PG3 major MSM subclade (86%), *S. sonnei* lineage III MSM clade 2 (89%), and sonnei-MSM-1 (80%) were lower compared to the total number of cases in these clusters (Figures 2, 3). Other isolates in these clusters were from men that reported not having had MSM contact or from women (Figures 2, 3). Most MSM-associated patients (79% of *S. flexneri* and 78% of *S. sonnei*) were diagnosed with shigellosis in the Amsterdam region, while the remaining MSM-associated patients were spread throughout Netherlands. Clusters *S. flexneri* PG3 major MSM subclade, flexneri-MSM-2, and *S. sonnei* lineage III MSM clade 4 contained both isolates from the Amsterdam region only. Clusters flexneri-MSM-2 and flexneri-MSM-3 were both distantly related to the reference *S. flexneri* PG3 minor MSM subclade, while flexneri-MSM-1 was not related to any of the MSM reference isolates (Figure 2). PrEP use was exclusively reported by patients infected with isolates located in the MSM clusters. HIV infections were mostly reported by patients in the MSM clusters, except for only 2 out of 19 patients infected with *S. flexneri* and one EIEC-infected patient (Figures 2–4). For patients related to MSM-associated *S. flexneri* clusters, the percentage of HIV infections or PrEP use ranged from 43% in the *S. flexneri* PG3 major MSM subclade cluster to 100% in the *S. flexneri* 3a MSM sublineage A cluster (Figure 2), while in the *S. sonnei* lineages III MSM clade 2 and MSM clade 4, 50% of patients had HIV or another STI and in cluster sonnei-MSM-1 this percentage was 30% (Figure 3). All MSM-related clusters contained isolates from both 2016 and 2017, indicating that these clusters were not restricted to a specific period. Additionally, all patients with isolates within the MSM clusters had no travel history or they had traveled within Europe (Figures 2, 3).

### Travel Associated Clusters

Travel-related clusters were present for *S. flexneri*, *S. sonnei* and EIEC (Figures 2–4). Most patients (80%) with *S. flexneri* serotype 6 reported travel to Africa. Three other small clusters of *S. flexneri* were travel-related; a cluster of two serotype 4av isolates linked to Africa, one cluster of two serotype1b isolates linked to Central America, and one cluster containing serotype Xv and a provisional *Shigella* was related to travel to South America (Figure 2). None of the *S. flexneri* isolates in our study were closely related to the travel-related references from 3a Africa and 3a Asia sublineages, nor were they restricted to certain time-periods within the 2 years of surveillance. For *S. sonnei*, three isolates that were distantly related to lineage IIIa reported travel to South America, the region to which lineage IIIa was associated (Baker et al., 2017; Figure 3). Four small *S. sonnei* clusters were related to travel to Central America (*n* = 4 to 8), four other small clusters (*n* = 2 to 9), and one large cluster (*n* = 22) were related to travel to Asia, and five clusters were related to travel to Africa (*n* = 4, 8, 13, 14, and 33). Furthermore, two out of the four clusters that were travel-related to Central America were from February to August 2016 and June to October 2016, respectively. For EIEC, two clusters were related to travel to Asia (*n* = 3, 5), one larger cluster (*n* = 9) was related to travel to Africa and one smaller cluster was related to South America (*n* = 4; Figure 4). The latter only contained isolates cultured from February to May 2016. Although other isolates were also travel-related, no other distinct clusters were found.

### Resistance Associated Clusters

Ciprofloxacin resistance was mainly observed in the flexneri-MSM-3 cluster, sonnei-MSM-1 cluster, and the *S. sonnei* Asian cluster (Figures 2, 3). These isolates possessed the three known chromosomal point mutations in the *gyrA* and *parC* genes. Moreover, isolates in the flexneri-MSM-3 cluster contained an extra point mutation in the *parE* gene that is also related to phenotypical ciprofloxacin resistance (Figure 2). The other isolates in a cluster related to flexneri-MSM-3 were also ciprofloxacin resistant, of which two isolates were from patients that reported travel to Asia and other isolates were from patients that reported no travel (Figure 2). Both azithromycin resistance genes were present in twenty *S. flexneri* and *S. sonnei* isolates and were only observed in MSM-clusters. A total of 18 of these isolates also displayed the *bla-TEM1b* gene, indicating the presence of the MSM-associated pKR S100 plasmid (Figures 2, 3). For EIEC, phenotypical AMR showed no specific cluster-related pattern. Overall, EIEC isolates were less resistant than *S. flexneri* or *S. sonnei* isolates (Figure 4).

### Virulence Profiling

In our study, none of the *Shigella* or EIEC isolates sequenced contained genes that encode for the Shiga-toxin *E. coli* virulence genes.

For *S. flexneri*, all but one isolate had the *set* gene located on the SHI-1 island. The *pic* gene was only present in *S. flexneri* 2a or Y, and the *sigA* gene was present in *S. flexneri* serotype 2a, Y, and 6 and with a lower identity percentage in *S. flexneri* serotype 3a and 3b (Figure 2). All isolates that possessed all genes present in the SHI-1 island were from PG3 (Figure 2). Isolates in the 3a MSM sublineage A cluster and *S. flexneri* serotype 6 possessed none of the *shi* genes in SHI-2. Three *S. flexneri* isolates lacked all genes encoding for the T3SS machinery and effectors (Figure 2). One isolate had the *Osp* genes, but lacked the *mxi-spa* operon, the *ipa-ipg* operon and the *virA* gene.

Almost all *S. sonnei* isolates had the *sigA* and *pic* genes from the SHI-1 island, while the *set* gene was present in approximately half of the isolates (Figure 3). All isolates had all genes present in the SHI-2 pathogenicity island, except for the *shiD* gene, which was present in only two isolates that clustered apart from other isolates in lineage III. More than half of the *S. sonnei* isolates did not own the genes encoding for the T3SS machinery and effectors (Figure 3).

In the analysis of virulence genes of the EIEC isolates, 54 isolates (84%) contained the *set* gene located on the SHI-1 island, all in combination with the *sen* (*ospD3*) gene encoded on the pINV plasmid (Figure 4). Ten EIEC isolates (16%) harbored no genes encoding for the T3SS machinery or effectors, of which three isolates also contained none of the genes present in the SHI-1 island (Figure 4). The other seven isolates contained the *sigA*, and/or the *pic* genes. The lineage that comprises isolates with ST6 and the lineage that comprises the ST99/O96 and ST4267 isolates did not contain SHI-2 or only a smaller number of genes present in this island. Only 11 EIEC isolates (17%) contained the *shiA* gene on this island, and none contained the *shiE* gene (Figure 4).

## Discussion

This study shows that *S. sonnei*, *S. flexneri*, and EIEC are the most prevalent *Shigella*/EIEC species in Netherlands. A substantial part of the collected *Shigella* spp. and EIEC isolates collected during the study is resistant to one or more of the first- and second-line antimicrobials for treatment. Identification with phenotypic methods and serotyping is challenging, as EIEC had no specific key characteristics and serotype switching is common in *S. flexneri* (The et al., 2016). Additionally, strains of MSM clusters from other countries were also identified among MSM-associated clusters in Netherlands, and those that were travel-related mostly clustered together. We confirm the overlap of MSM-associated clusters with patients that reported HIV infection and with AMR to azithromycin and ciprofloxacin. Moreover, isolates from domestically acquired infections sometimes belonged to travel-related clusters, indicating secondary transmission of imported isolates.

Phenotypic characteristics of the pathotype EIEC were described based on 64 isolates in this study. If EIEC isolates display one of the phenotypic characteristics that are by definition negative for *Shigella* spp., the distinction is uncomplicated. In contrast, when EIEC isolates display the more inactive *Shigella* phenotype, distinction is challenging (van den Beld and Reubsaet, 2012). Identification and distinction of *Shigella* spp. and EIEC is not always possible, even with the thorough phenotyping and serotyping that was performed. Because of their relatedness, other commonly used techniques for microbiological identification, as MALDI-TOF mass spectrometry and molecular detection of species-specific genes, cannot distinguish *Shigella* and EIEC (Hale, 1991; van den Beld and Reubsaet, 2012). One isolate in our study could not be assigned to the genus *Shigella* or *Escherichia* and ten *Shigella* isolates could not be assigned to a species. Moreover, in the cgMLST tree combining all species, clusters with multiple species were formed. This was most likely due to deviating phenotypic features or inconclusive serotypes that influenced their identification. All provisional *Shigella* isolates lacked specific serological characteristics. Therefore it is impossible to determine the species, but they clustered mostly with *S. flexneri* (Figure 1). Six of the eight EIEC isolates that clustered within *S. flexneri* fitted phenotypically EIEC as well as *S. flexneri*. However, they all had an inconclusive *Shigella* serotype (Figure 1). The *E. coli* somatic antigen type for these six isolates was O135, known to be EIEC-associated and also known to have cross-reactions with multiple *S. flexneri* serotypes (DebRoy et al., 2016). Taken the phylogeny into account, it is plausible that these isolates are, in fact *S. flexneri*, but for an unknown reason, they lack to display parts of the serological features. The two remaining EIEC isolates within *S. flexneri* had multiple phenotypical characteristics that do not fit the description of *Shigella*, e.g., motility and LDC production. Additionally, one EIEC isolate clustered within *S. sonnei*, and seems to be a hybrid isolate. Phenotypically, it is classified as EIEC because the isolate is motile and produces indole and gas from glucose. However, based on the serotype it can be classified as *S. sonnei*. One *S. flexneri* serotype 2a isolate clustered with EIEC. Phenotypically, it could be classified as both *S. flexneri* and EIEC. However, it did not have the *E. coli* somatic antigen type O1 until O188. These mixed-species clusters with inconclusive serotypes and hybrid isolates confirms the close genetic relationship among the species of *Shigella* and EIEC that was described before in multiple studies (Kaper et al., 2004; Lan et al., 2004; Pettengill et al., 2015; Hazen et al., 2016).

The large diversity of EIEC isolates in the United States (Pettengill et al., 2015) was also confirmed in Netherlands, as diverse *E. coli* O-types and Warwick MLST types were found to be circulating. Additionally, in the cgMLST, EIEC isolates showed more diversity than *S. flexneri* or *S. sonnei*.

Shigella *flexneri* and EIEC isolates clustered mostly according to their serotype in the cgMLST. An exception were two *S. flexneri* Yv isolates forming a separate cluster probably since they relate to the different phylogroups PG1 and PG6 as shown in the cgMLST tree (Figure 2). It was described that serotypes can belong to multiple PGs, although the association of *S. flexneri* Yv with PG1 was not found before (Connor et al., 2015). However, because *S. flexneri* can switch their serotype due to the exchange of O-antigen genes via horizontal gene transfer (HGT; The et al., 2016), a plausible hypothesis is that the more isolates are sequenced, the more serotypes per PG will be found. The clustering of five O164 isolates with reference EIEC genome ST270/O124 can be explained by the strong resemblance between O164 and O124 antigens (31). Although isolates cluster roughly on serotype-level and serotyping is used for the description of individual isolates, some serotypes form multiple clusters and serotype switching is common. Therefore, techniques with a higher resolution as WGS provide more information for communication and surveillance purposes or outbreak investigations.

Without support of bacterial typing, contact tracing and outbreak investigations amongst the MSM population in particular can be complicated due to high numbers of sexual partners and anonymous sex, making it difficult to establish epidemiological links between cases (Gilbart et al., 2015; Pijnacker et al., 2017). With our study, we proved that isolates related to international MSM-clusters are circulating in Netherlands. Additionally, we found one MSM-associated *S. sonnei* cluster and three MSM-associated clusters in *S. flexneri* not related to international reference isolates that were included here. One of the clusters consisting of Dutch isolates only, contained only *S. flexneri* serotype 1c, and to our knowledge, this study is the first that associates *S. flexneri* 1c with the MSM population. The fact that in our study, MSM-associated *S. flexneri* and *S. sonnei* clusters also contained isolates from men that reported no sexual contact with other men or isolates from women, could indicate spillover to the non-MSM population, or (partially) due to misclassification of MSM as non-MSM. The allocation of isolates from 2016 and 2017 to all *S. flexneri* and *S. sonnei* MSM clusters provides evidence for prolonged circulation of these (inter)nationally MSM-associated *Shigella* isolates in Netherlands.

Outbreak investigations and other surveillance studies have indicated a large overlap between shigellosis amongst MSM and HIV (Mohan et al., 2018; Ingle et al., 2019). This was confirmed by our study for the Dutch situation. This coexistence of shigellosis amongst MSM and HIV is thought to have multiple causes, as specific sexual practices, substance use or the use of social media that might cause serosorting based on HIV status, enhanced by increased shedding of bacteria due to high numbers of multidrug resistance (Mohan et al., 2018; Ingle et al., 2019).

While 97% of MSM-associated shigellosis cases were domestic or acquired from travel to other European countries only, 71% of shigellosis cases in the non-MSM population were related to travel outside of Europe. Clusters related to travel were displayed in *S. flexneri* as well as *S. sonnei*. For EIEC, limited data on travel history for patients was available. Within the clusters related to travel, also domestically acquired isolates were present, indicating secondary transmission of imported isolates in Netherlands.

In Dutch guidelines, cotrimoxazol, ciprofloxacin and azithromycin are advised for treatment of shigellosis cases (SWAB, 2014). Azithromycin was not tested by any of the laboratories, because clinical breakpoints are not known from EUCAST guidelines (EUCAST, 2019). However, *in silico* determination of azithromycin resistance genes *erm(B)* and *mphA* revealed the presence of azithromycin resistance in isolates from Netherlands. In a vast majority of the isolates in which both azithromycin resistance genes were present, the *bla-TEM1b* gene was also present. This combination of genes was only observed in isolates within the MSM clusters. All these genes were described to be present on the pKSR100 plasmid that is associated with HGT within MSM lineages before (Baker et al., 2018b). Our study confirms the association of ciprofloxacin resistance with isolates from MSM and travel to Asia (Chung The et al., 2016; Ingle et al., 2019). Furthermore, the resistance to advised treatments cotrimoxazol, ciprofloxacin and azithromycin was present throughout the collection period in our dataset, and was predominantly lineage-specific, confirming earlier observations that the acquirement of ARGs through HGT drives the epidemiological outcomes and success of certain lineages (Holt et al., 2012; Baker et al., 2018b).

Our study confirmed observations made earlier in *E. coli* and *S. sonnei*, that correlation of detected ARGs to phenotypic outcome is significant, except for the aminoglycosides (Stoesser et al., 2013; Zankari et al., 2013; Tyson et al., 2015; Sadouki et al., 2017). Although none of the gentamicin and tobramycin susceptible isolates contained one of the *aac(3)-IId* or *aph(3)Ia* genes, only low percentages of resistant phenotypes had one or more of these genes. Presumably, another resistance mechanism not identified by the methods used in our study causes resistant phenotypes, which requires further investigation. Additionally, our study confirmed that the presence of two or more chromosomal point mutations in the *gyrA* and *par* genes was significantly associated with phenotypic ciprofloxacin resistance, while the presence of the plasmid-mediated *qnr* genes or only one chromosomal point mutation, predominantly *gyrA* S83L, was not significantly associated with phenotypic resistance to ciprofloxacin (Chung The et al., 2016). The presence of point mutation *gyrA* S83L was thought to be a precursor for the full ciprofloxacin resistant phenotype, requiring at least one additional chromosomal point mutation (Chung The et al., 2016; Sadouki et al., 2017).

Almost all *S. flexneri* and EIEC isolates possessed virulence genes present in the pINV plasmid, while these genes were only detected in approximately half of the *S. sonnei* isolates. It is known that in *S. sonnei*, the pINV plasmid is frequently lost during subculturing (The et al., 2016). *S. flexneri* and EIEC isolates were present that lacked the genes encoding for the T3SS machinery and effectors. This is probably due to the excision of parts of the T3SS region. This phenomenon was described before and is thought to result from the high fitness costs of this region for the bacteria while being outside the human host (Pilla et al., 2017). In an earlier study, the presence of virulence genes in EIEC isolates was described (Hazen et al., 2016). Compared to our study, we examined some differences. First, in our study, almost all (84%) EIEC isolates contained the *set* gene, in contrast to the 15% of EIEC isolates described in the earlier study (Hazen et al., 2016). Second, in our study, EIEC isolates containing the *shiA* gene in the SHI-2 island were observed, while the earlier study described this gene as absent from all EIEC (Hazen et al., 2016). These differences can be explained because different isolate sets were used from different geographical origins. Another observation from our study is that some lineages of EIEC did not have the SHI-2 pathogenicity island at all, which seems to be lineage specific. They might possess another pathogenicity island, containing genes involved in the same processes as the genes located on SHI-2 in *S. flexneri* and *S. sonnei*. Another explanation could be that these EIEC isolates are precursors of *Shigella* spp. and are in transition to gain full virulence potential as hypothesized earlier (Lan et al., 2004). Nonetheless, these EIEC isolates were capable of causing disease, because all isolates were collected from patients with symptoms. From 72% of these patients EIEC was the only detected pathogen (van den Beld et al., 2019b).

A strength of this study is that we combined microbiological characteristics of *Shigella* spp. and EIEC isolates with detailed epidemiological data of the patients. In addition, our study is representative for the *Shigella* spp. and EIEC isolates in Netherlands, as they were collected from MMLs geographically distributed over the country.

Limitations of this study are that epidemiological data was collected by interviewing patients, and was therefore not an objective measurement. Although this probably does not have a major effect on the reported sexes of patients or travel history, MSM contact and HIV or STI status might be underreported. Furthermore, for EIEC isolates the cluster formation was not as distinct as for *S. flexneri* and *S. sonnei*, probably due to the diversity of the isolates and to limited availability of epidemiological data. Moreover, as in only half of the shigellosis cases an isolate can be obtained (de Boer et al., 2010; Liu et al., 2016; Van Lint et al., 2016) and not all *Shigella* spp. and EIEC isolates detected in Netherlands in 2016 and 2017 were available for this study, the observed clusters probably were substantially larger, and some clusters might have been missed.

In Netherlands, thorough shigellosis case investigations are routinely performed, which results in a comprehensive knowledge of epidemiological data. However, the current guidelines, in which no laboratory surveillance of *Shigella* spp. is conducted, are not sufficient to detect all national and international clusters due to the low resolution of serotyping and due to the challenging contact investigations of MSM groups in particular. This study emphasized that epidemiological and laboratory surveillance are complementary to each other. Furthermore, multifactorial public health approaches for (inter)national surveillance purposes and outbreak investigations are important, particularly when combined with thorough characterization of isolates using techniques with high discriminatory power such as WGS. Our study was a snapshot in time, but it is important to monitor these (inter)national patterns for *Shigella* spp. over longer periods to enable outbreak detection, following improved prevention and targeted responses by public health authorities.

## All Members of the IBESS Working Group

The IBESS group provided isolates and patient data, and consists of the following contributors from Netherlands: M.J.C. van den Beld, National Institute for Public Health and the Environment (RIVM), Centre for Infectious Disease Control, Bilthoven and Department of Medical Microbiology and Infection Prevention, University of Groningen, University Medical Center Groningen, Groningen. E. Warmelink, Public Health Service GGD Groningen, Groningen. A.M.D. Kooistra-Smid, Certe, Department of Medical Microbiology, Groningen and Department of Medical Microbiology and Infection Prevention, University of Groningen, University Medical Center Groningen, Groningen. A.W. Friedrich, Department of Medical Microbiology and Infection Prevention, University of Groningen, University Medical Center Groningen, Groningen. F.A.G. Reubsaet, National Institute for Public Health and the Environment (RIVM), Centre for Infectious Disease Control, Bilthoven. D.W. Notermans, National Institute for Public Health and the Environment (RIVM), Centre for Infectious Disease Control, Bilthoven. M.W.F. Petrignani, Public health service GGD Amsterdam, Amsterdam. T. Waegemaekers, Public health service GGD Gelderland-Midden, Arnhem. J.W.A. Rossen, Department of Medical Microbiology and Infection Prevention, University of Groningen, University Medical Center Groningen, Groningen. A.P. van Dam, Amsterdam Health Service, Amsterdam. S. Svraka-Latifovic, CBSL, Tergooi, Hilversum. J.J. Verweij, Elisabeth-TweeSteden Hospital, Laboratory for Medical Microbiology and Immunology, Tilburg. L.E.S. Bruijnesteijn van Coppenraet, Isala, Laboratory for Medical Microbiology and Infectious diseases, Zwolle. K. Waar, Izore, Centre for Infectious Diseases Friesland, Leeuwarden. M. Hermans, Jeroen Bosch Ziekenhuis, Laboratorium Medische Microbiologie, ‘s-Hertogenbosch. D.L.J. Hess, LabMicTA, Laboratory for Medical Microbiology and Public Health, Hengelo. L.J.M. van Mook, Microvida location Amphia, Breda. M.C. Bergmans, Microvida location Bravis, Roosendaal. R.R. Jansen, OLVG, Medical Microbiological Laboratory, Amsterdam. J.H.B. van de Bovenkamp, PAMM Laboratory for Medical Microbiology, Veldhoven. A. Demeulemeester, SHL-group, Etten-Leur. E. Reinders, St. Antonius Ziekenhuis, Medical Microbiology and Immunology, Nieuwegein. F.M. Linssen, Zuyderland Medical Centre, Medical Microbiology, Heerlen. All adjacent Public Health Services.

## Data Availability Statement

Publicly available datasets were analyzed in this study. This data can be found here: https://www.ebi.ac.uk/ena/browser/view/PRJEB32617.

## Ethics Statement

Ethical review and approval was not required for the study on human participants in accordance with the local legislation and institutional requirements. Written informed consent for participation was not required for this study in accordance with the national legislation and the institutional requirements.

## Author Contributions

MB, FR, AK-S, and JR conceptualized the project and designed experiments. AH, SK, EH, BH-B, RN, and ACAH performed experiments. MB, DB, and HH analyzed the data. MB, RP, FR, AK-S, and JR interpreted results. FR, AK-S, and JR supervised the project. MB wrote the manuscript. All authors read, reviewed, and approved the final manuscript. All authors contributed to the article and approved the submitted version.

## Conflict of Interest

JR is currently employed by IDbyDNA Inc. The remaining authors declare that the research was conducted in the absence of any commercial or financial relationships that could be construed as a potential conflict of interest.

## Footnotes

1 https://github.com/Papos92

2 https://bitbucket.org/genomicepidemiology/resfinder/src/master/

## Supplementary Material

Supplementary Table 1 Details and accession numbers of used reference genomes. In this file, the accession numbers of the used reference isolates are depicted, including their details about assigned species and types by the original studies.

## Abbreviations

AMR: Antimicrobial resistance
ARG: Antimicrobial resistance gene
CGE: Center for Genomic Epidemiology
cgMLST: Core genome multi-locus sequence typing
CI: Confidence Interval
DNA: Deoxyribonucleic acid
EIEC: entero-invasive *Escherichia coli*
HGT: Horizontal gene transfer
HIV: Human Immunodeficiency Virus
IBESS: Invasive Bacteria *E. coli-Shigella* Study
MALDI-TOF: matrix-assisted laser desorption/ionization- time of flight
MGE: mobile genetic elements
MLST: multi-locus sequence typing
MMLs: medical microbiological laboratories
MSM: men who have sex with men
OR: odds ratio
PCR: polymerase chain reaction
PG: phylogroup
pINV: large invasion virulence plasmid
PrEP: pre-exposure prophylaxis
SHI: *Shigella* island
Spp.: Species
SRA: Sequence Read Archive
ST: sequence type
STI: sexually transmitted infection
T3SS: Type III secretion system
WGS: whole genome sequencing.
